# Development of a Medical Device in Response to a Fatal Self-Injection of Non-prescribed Opioids: A Case Report

**DOI:** 10.7759/cureus.56869

**Published:** 2024-03-25

**Authors:** Kara Bragg, Michael Albus, Leslie V Simon, Bradley Bragg, Rachelle Beste

**Affiliations:** 1 Emergency Medicine, Mayo Clinic, Jacksonville, USA; 2 Emergency Medicine/Medical Simulation, Mayo Clinic, Jacksonville, USA

**Keywords:** line infections, self-injection of non-prescribed substances into vascular devices, patients who inject drugs, case study, overdose, vascular access device, opioid use disorder

## Abstract

Patients who inject drugs (PWID) pose unique challenges in their medical care due to risks of increased infection and overdose. There are no known commercially available devices to prevent patients from self-injecting non-prescribed substances into vascular access devices (VADs). A patient in the emergency department (ED) of a midsized suburban hospital self-injected an opioid in the ED restroom after the placement of a vascular catheter by the nursing staff as part of her ED care. Despite precautions taken for a patient with a known opioid use disorder (OUD) and a history of self-injecting non-prescribed substances into VADs, the patient suffered a self-induced fatal overdose. PWID are at significant risk of self-injection when requiring intravenous medications as part of their medical care. This case highlighted the need for formal reporting for patients who self-inject non-prescribed substances into VADs. It revealed a lack of medical devices to help providers ensure that PWID cannot access their medical devices when intravenous therapy is indicated.

## Introduction

The opioid epidemic has increased dramatically since 1999 leading to one of the United States' top healthcare crises [1]. Since the Covid-19 pandemic and the time of this case study, the rates have increased by 15% from 2020 to 2021 [2]. Efforts have focused on restricting the opioid prescription supply, but this does not address the needs of the patients requiring intravenous therapy [3]. Patients who inject drugs (PWID) are at greater risk for complications with a vascular access device (VAD) including bloodstream infections, line infections, bacteremia, endocarditis, overdose, and death [4-6]. The costs associated with the complications of self-injection of non-prescribed substances into vascular devices (SIVAD) are associated with increases in length of stay (LOS) readmissions and increased healthcare costs [6,7]. Patients are more prone to leave against medical advice (AMA) secondary to intentional withholding of intravenous access. There are no known commercially available devices to prevent patients from SIVAD [8].

The opioid epidemic has caused an increase in hospitalizations associated with opioid use disorder (OUD) [9]. PWID may require intravenous medications during their treatment. This poses a dilemma for both the patient and the provider. PWID have a higher risk of self-injecting during a time when they may be experiencing withdrawal or cravings [4,7,10]. Medical providers must balance the risks of VAD and the risk of SIVAD by the patient leading to additional complications [7]. Nurses must endure the cognitive burden of managing at-risk patients with unprotected VADs while providing the parental therapy necessary for appropriate treatment [10]. 

Furthermore, PWID is often at increased risk of poor outcomes secondary to other confounding factors such as lack of insurance, psychiatric disorders, and other forms of substance abuse [5]. Providers are often reluctant to discharge patients home on parenteral therapy for fear of SIVAD leading to subsequent prolonged hospital stays [9]. Patients are subject to bias and stigma during their care [5,11]. Furthermore, PWID is a high-risk population, often leaving AMA leading to incomplete treatment leading to increased morbidity, readmission, and death [5].

At the time of our patient’s death, there were few options available to assist with the management of PWID in the hospital setting. Direct observation, detection devices, or removing the access site between infusions were the standards of care [10]. Furthermore, there was a lack of reported evidence regarding the risks associated with a PWID and receiving intravenous therapy in either the inpatient or outpatient setting [4]. Studies published since our initial research reported up to 40% of OUD patients with SIVAD intended for antimicrobial use [5].

## Case presentation

A Caucasian female patient in her mid-forties presented to the emergency department (ED) with right breast tenderness, fever, chills, “rigors,” nausea, vomiting, and generalized malaise. Her medical history was significant for opioid dependence, mastocytosis, multiple myeloma, and recurrent cellulitis and abscess in the breast. The patient was seen in the ED multiple times that year and had been treated recently for sepsis. The patient met inpatient criteria for acute kidney injury and received IV cefazolin for recurrent infection with infectious disease consult. Before arrival in the ED, the patient was prescribed a 100 mg fentanyl patch in place and up to 12 mg of hydromorphone every four hours for chronic pain from her mastocytosis. The patient also required diphenhydramine before antibiotic administration to prevent itching. She received two 12.5 mg doses of diphenhydramine on her precedent of needing anti-pruritic treatment before cefazolin. She also received 12.5 mg of promethazine. 

The patient was intentionally roomed across from the nursing station, and all her belongings were placed at the glass door entrance to her room. After she was admitted to the hospital, she was held in the ED for 3.5 hours waiting for an inpatient bed. Before her transfer, she requested to use the restroom. After she failed to return to her room, the staff performed a check and found her unresponsive on the bathroom floor with a syringe attached to her VAD. She underwent resuscitation unsuccessfully and was pronounced dead. 

## Discussion

This case highlights the conundrum that healthcare providers face when caring for PWID. Patients are at increased risk of infection, overdose, and death while adding to the duration cost of their healthcare [6,7]. Despite precautions taken, the patient found an opportunity to use her own VAD, which caused a fatal overdose. SIVAD is likely during a hospital stay unless the underlying issue of OUD is addressed [5]. The dilemma arises on how to adequately provide analgesia safely in the hospital setting as most nurses and providers understand that placing a VAD allows SIVAD [10]. A literature review on the topic found a lack of publications on the prevalence and statistics regarding the frequency of SIVAD at the time of this case. Recent studies show an increasing acknowledgment of the need for formal management [6,7]. There was a lack of comprehensive protocols defining the management of patients in the hospital and outpatient settings [7]. Current methods in use were direct supervision, video surveillance detection tape, and protective caps that needed to be removed for therapy. There was no known medical device that would prevent a patient from accessing their device while still making it accessible to the provider [8]. This prompted the design of a multiuse medical device that would allow patients to have intravenous access, intermittent and continuous infusions, and well as piggy-backed medications (Table 1).

**Table 1:** Comparison table of systems currently in use versus IV SafeLock as a means to monitor IV access sites on patients IV, intravenous

	No device	Neuma clamp	Tamper evident lock	Direct 1:1 supervision	Video monitoring	IV SafeLock
Device cost	√	√	√	X	X	√
Ease of use	√	√	√	X	X	√
Protection	X	X	X	√	√	√
Secure and reusable	X	X	X	√	√	√
Useable on all access ports	√	√	√	√	√	√

**Figure 1:**
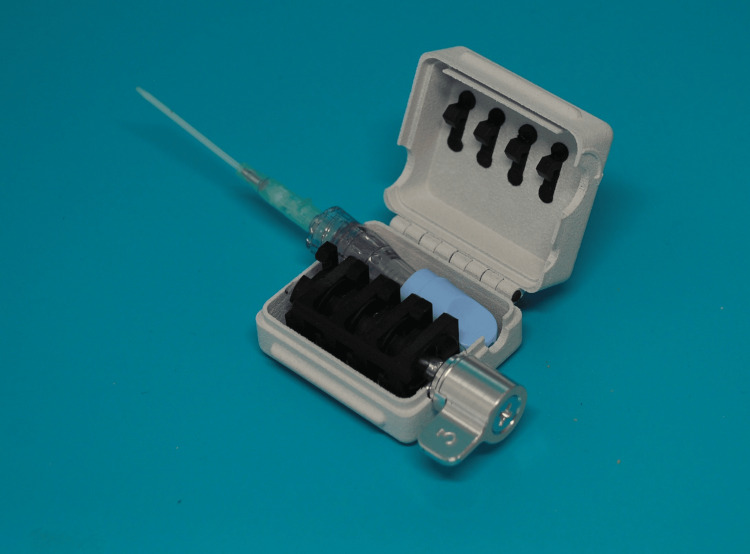
IV SafeLock prototype shown with INT catheter in place, key inserted, and in the open unlocked position INT, intermittent needle therapy

The device surrounds all infusion ports and hubs allowing access by nurses for medication administration yet preventing access by the patient (Figure 1). 

A clinical trial showed the device to be functional in an ED setting, protecting the infusion ports while allowing intravenous therapy [8]. The device may alleviate fears that providers have expressed about placing VADs in PWID [10]. The device would also potentially allow for outpatient parenteral antimicrobial therapy often limited secondary to fear of SIVAD by patients with OUD [4,11]. VAD protection can help keep medical costs down by preventing line infections, endocarditis, prolonged hospitalization, and patients leaving AMA [5].

## Conclusions

This case study, while not unique, highlighted deficits in the healthcare industry for managing PWID who require intravenous therapy in the acute care and home health setting. There was a lack of information available regarding the reporting and frequency of SIVAD. Reasons could include fear of lower scores for the institution on benchmarking. Second, there was a distinct lack of equipment that would allow providers to safely address the need for intravascular access in PWID. This case prompted the development of a medical device that will allow providers to safely provide intravenous therapy in PWID. The device proved successful in its first clinical trial in the ED setting. This case also shows a need for more comprehensive, non-punitive reporting by hospitals of SIVAD to understand the scope of the problem and allow institutions to create protocols and use protective equipment to minimize adverse patient outcomes in this population. 
